# 

**Published:** 1896-03

**Authors:** 


					﻿PHOSPHO-MURIATE of QUININE, comp. (PHILLIPS’.)
IN DEFICIENCY OF THE PHOSPHATES, LACK OF NERVE TONE, MALARIAL M AN I FEjJXAX Wl^g,
CONVALESCENCE FROM THE EXANTHEMATA, ETC.—WILL NEVER DISAPPOINT.	/?/-•*
BEWARE OF THE MANY IMITATIONS.
Insist on PHILLIPS’» <
THE CHAS. H. PHILLIPS CHEMICAL CO., 77 Pine Sr. fgç<yo<K.
Southern Medical Record.
A Monthly Journal of Medicine and Surgery.
____________________________________' • /
Vol. XXVI.
ATLANTA, GA., MARCH, 1896.
No. Ill-
BERNARD WOLFF, M. D., Editor.
LOUIS H. JONES, M. D., Business Manager.
Associate Editors:
A. W. GRIGGS, M. D.
WILLIS F. WESTMORELAND. M. D.
j. McFadden gastón, m. d.
Entered at the Post Office at Atlanta, Ga., as Second-Class Matter.
The Foote & Davies Co., Atlanta, Ga.
				

